# Uptake and adoption of the NHS App in England: an observational study

**DOI:** 10.3399/BJGP.2022.0150

**Published:** 2023-10-03

**Authors:** Sukriti KC, Salina Tewolde, Anthony A Laverty, Céire Costelloe, Chrysanthi Papoutsi, Claire Reidy, Bernard Gudgin, Craig Shenton, Azeem Majeed, John Powell, Felix Greaves

**Affiliations:** Department of Primary Care and Public Health, Imperial College London;; Department of Primary Care and Public Health, Imperial College London;; Department of Primary Care and Public Health, Imperial College London;; Institute of Cancer Research, London.; Nuffield Department of Primary Care Health Sciences, University of Oxford, Oxford;; Nuffield Department of Primary Care Health Sciences, University of Oxford, Oxford;; Patient and public involvement representative and a member of the University of Oxford Advanced Research Computing board;; NHSX Analytics Unit, NHS England;; Department of Primary Care and Public Health, Imperial College London;; Nuffield Department of Primary Care Health Sciences, University of Oxford, Oxford.; Department of Primary Care and Public Health, Imperial College London, UK.

**Keywords:** patient portals, electronic health records, general practice

## Abstract

**Background:**

Technological advances have led to the use of patient portals that give people digital access to their personal health information. The NHS App was launched in January 2019 as a ‘front door’ to digitally enabled health services.

**Aim:**

To evaluate patterns of uptake of the NHS App, subgroup differences in registration, and the impact of COVID-19.

**Design and setting:**

An observational study using monthly NHS App user data at general-practice level in England was conducted.

**Method:**

Descriptive statistics and time-series analysis explored monthly NHS App use from January 2019–May 2021. Interrupted time-series models were used to identify changes in the level and trend of use of different functionalities, before and after the first COVID-19 lockdown. Negative binomial regression assessed differences in app registration by markers of general-practice level sociodemographic variables.

**Result:**

Between January 2019 and May 2021, there were 8 524 882 NHS App downloads and 4 449 869 registrations, with a 4-fold increase in App downloads when the COVID Pass feature was introduced. Analyses by sociodemographic data found 25% lower registrations in the most deprived practices (*P*<0.001), and 44% more registrations in the largest sized practices (*P*<0.001). Registration rates were 36% higher in practices with the highest proportion of registered White patients (*P*<0.001), 23% higher in practices with the largest proportion of 15–34-year-olds (*P*<0.001) and 2% lower in practices with highest proportion of people with long-term care needs (*P*<0.001).

**Conclusion:**

The uptake of the NHS App substantially increased post-lockdown, most significantly after the NHS COVID Pass feature was introduced. An unequal pattern of app registration was identified, and the use of different functions varied. Further research is needed to understand these patterns of inequalities and their impact on patient experience.

## INTRODUCTION

The increasing adoption of electronic medical records (EMRs) by hospitals and general practices have given patients digital access to their personal health information through the utilisation of patient portals. Patient portals provide the potential for improved patient–provider communication via electronic messaging, direct appointment booking, prescription ordering, and provide users with a platform to be more informed about their health.^1^

Although some preliminary research has shown that patient portals may improve health outcomes, their direct benefits are still unclear.^2^^,^^3^ Studies have highlighted an overall positive impact of using these technologies, but issues around patient confidentiality, security, and trust remain.^1^^,^^4^^,^^5^

There are also reports of unintended consequences of online access to health records with impacts on patient autonomy, documentation practices, and increased workload, which affect patients and healthcare staff.^6^ Recent reviews and national surveys on patient portals have shown their widespread use in other countries; and that patients’ interest and ability to use them is influenced by factors such as age, gender, ethnic group, health literacy, and health status.^7^^–^^9^ However, there are uncertainties surrounding the impact on these portals on existing health inequalities, patient experience, and clinical outcomes.^10^

### The NHS App

In January 2019, the NHS in England introduced a new app for patients called the NHS App, which offered the following functions at launch for general use:

1. check symptoms using NHS 111 online and the health A-Z on the NHS website;
2. view their general practice medical record;
3. book and manage appointments at their general practice;
4. order repeat prescriptions;
5. register as an organ donor; and
6. set data sharing preferences for the national data: choose if data are shared for planning and research.

**Table:** How this fits in

Research has shown that patient portals may improve health outcomes. However, there are known issues around confidentiality and growing concerns that they may be contributing to, worsening, or even creating new health divides. This study found that the uptake of the NHS App in England was positive but driven by COVID-19-related events. App registration was unequal between different population groups and there was a varied pattern of use of the different functions. Research is needed to understand how the NHS App influences health equity and patient outcomes.

In light of the novel coronavirus (COVID- 19) pandemic, a new feature called the NHS COVID Pass was launched on 17 May 2021,^11^ which allowed users to prove their vaccination status. NHS England’s goals for the app were to: 1) improve access to primary care services; 2) improve patient experience; 3) save time in general practices; and 4) promote self-care.

This research aimed to evaluate the effectiveness of the NHS App in meeting its goals and to explore the trends of uptake by looking at early patterns of app adoption across different population groups and in relation to recent time trends.

## METHOD

### Data

Data from the NHS App were analysed using data metrics from the NHS App dashboard.^12^ The NHS App dashboard was developed by NHS England, NHS Improvement, and NHS Digital under England’s national healthcare system. The organisations have now merged into a single organisation, and the NHS App dashboard is now provided by NHS England. Metrics currently available through the dashboard include aggregate counts of the number of registrations, downloads, appointment bookings and cancellations, GP health records viewed, prescription requests, visits to NHS 111 online, organ donation registrations and withdrawals, visits to the health A–Z page, and to the national data opt-out page. The research team received monthly aggregated data from the NHS App team at NHS Digital and did not have access to any personal or personally identifiable information. All NHS App metrics and descriptions are included in Supplementary Table S1.

Covariates data mapping sociodemographic profile of patients at the general-practice level were obtained from several verifiable public health data sources. Sociodemographic data on the age and gender of all GP-registered population were obtained from the NHS Digital website.^13^ Data on ethnic composition and deprivation were obtained from Fingertips public health profile,^14^ and data on long-term health conditions were extracted from the GP Patient Survey (GPPS) database.^15^ (Supplementary Table S2).

### Analysis

#### Descriptive statistics

The study period for this analysis was from January 2019–May 2021. Descriptive statistics were used to summarise monthly NHS App metrics at a general-practice level nationally. Absolute monthly counts were presented from January 2019–May 2021 of total app downloads, registrations, login sessions, appointments booked, GP health records viewed, and prescriptions ordered.

#### Time-series analysis

An ecological interrupted time-series (ITS) analysis was used to analyse the impact of the first UK national lockdown due to COVID-19 that occurred on 26 March 2020. The time-series analysis was used to evaluate changes in uptake and the longitudinal impact of the pandemic on different functionalities of the app.^16^ An interruption was added to the time series on 1 April 2020, to estimate changes in uptake before and after announcement of the first lockdown. The ITS explored changes in level and trend for national app logins, appointment bookings, GP health records viewed, and prescriptions ordered between January 2019 and May 2021.

The ITS model was then used to assess if there was a change in app usage immediately after the first lockdown and if a change in trend occurred over the whole study period. Absolute and relative changes of the post- intervention trend were also estimated, had the first lockdown not taken place. The absolute change in trend was calculated by taking the difference between the predicted pre-lockdown trend of the outcome and the post-lockdown trend at the end of the study period. The relative change was calculated as the absolute change as a relative proportion. To analyse app usage and uptake before the NHS COVID Pass was introduced, a separate ITS analysis was conducted excluding the month of May 2021. Autocorrelation was assessed by analysing the residuals of the ITS models^16^ (Supplementary Table S3).

#### Cross-sectional analysis

A cross-sectional analysis of the differences in NHS App registration rates at February 2021 was conducted across categories of age, sex, ethnic group, Index of Multiple Deprivation (IMD), size of the general practice, and long- term physical or mental health conditions, disabilities or illness. A negative binomial regression model using incident rate ratios (IRR) was applied to explore variations in NHS App registrations across these groups. For analysis, the sociodemographic covariates were ranked into four quartiles using the following identifiers: age (percent aged 15–34 years), gender (percent males), ethnic group (percent White), chronic care needs (percent with long-term healthcare needs), and practice size (total number of GP-registered patients aged >15 years). Quartile 1 included the lowest ranked practice and quartile 4 the highest. The only exception to this was the IMD rank that followed the Office for National Statistics (ONS) method of grouping into quintiles.^17^ Quintile 1 included practices with the lowest IMD score and quintile 5 included practices with the highest IMD score. Cumulative total NHS App registration rate per 1000 GP-registered population at February 2021 was considered as the outcome variable.

The Strengthening the Reporting of Observational Studies in Epidemiology (STROBE) guidelines were used for the reporting and analysis of this study.^18^

## RESULTS

### NHS App registrations and downloads

**Figure 1a.**
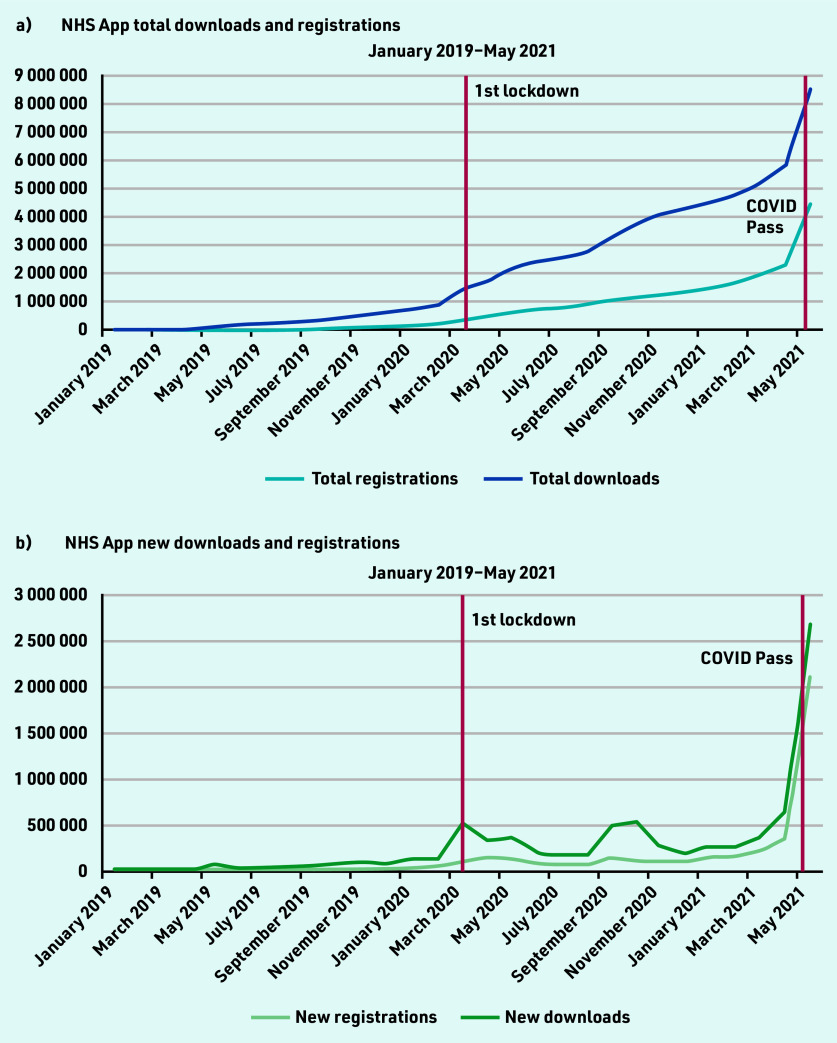
*Monthly cumulative NHS App registrations and downloads. Figure 1b. Monthly new NHS App registrations and downloads.*

There were 8 524 882 NHS App downloads and 4 449 869 registrations from January 2019–May 2021 (Figure 1a and 1b). At the start of the study period, there were 31 633 downloads and 1279 registrations. At the onset of the first UK lockdown in March 2020, there was a spike of 532 275 app downloads. The highest number of downloads occurred after the announcement and launch of the NHS COVID Pass in May 2021, with a total of 2 668 535 downloads.

There was a 4-fold increase in app downloads from April 2021 (650 558 downloads) to May 2021 (2 668 535 downloads) when the COVID Pass feature was introduced. During this month, 2 099 234 users registered for the app. At the end of May 2021, there were 51 956 423 GP-registered patients in England. Of these registered patients, 8.6% (*n* = 4 449 869) of the population aged ≥13 years were registered for the NHS App.

### Subgroup differences in app registration

The negative binomial regression model measured a difference in the NHS App registration rate, measured for each of the covariates with others held constant. The IRR comparing the practices in the highest versus the lowest quantile for each variable found 25% lower registrations in the most deprived practices (IRR 0.75, 95% confidence interval [CI] = 0.74 to 0.75; *P*<0.001), and 44% higher registrations in the largest-sized practices (IRR 1.44, 95% CI = 1.42 to 1.45; *P*<0.001).

App registration was 36% higher in practices with the highest percentage of White patients (IRR 1.36, 95% CI = 1.34 to 1.38; *P*<0.001) and 23% higher in practices with the highest percentage of patients aged 15–34 years (IRR 1.23, 95% CI = 1.21 to 1.25; *P*<0.001). Practices with the highest percentage of males had 13% lower registrations (IRR 0.87; 95% CI = 0.87 to 0.88; *P*<0.001), which was also 2% lower in practices with the highest percentage of people with long-term care needs (0.98, 95% CI = 0.97 to 0.99; *P*<0.001), when compared to the reference groups (Table 1).

**Table 1.** Negative binomial regression table showing unit change in the NHS App registration rate per 1000 GP-registered population, with other variables held constant, calculated using a negative binomial regression model

**Registration rate/1000**	**% Difference^a^**	**IRR**	***P*-value**	**95% Confidence interval**
**IMD quintile**				
(Reference group 1 = least deprived practices)				
2	−6	0.94	<0.001	0.93 to 0.95
3	−8	0.92	<0.001	0.91 to 0.93
4	−9	0.91	<0.001	0.90 to 0.92
5 (Most deprived practices)	−25	0.75	<0.001	0.74 to 0.75

**Percentage males**				
(Reference group 1 = practices with the lowest percentage of males)				
2	−11	0.89	<0.001	0.89 to 0.90
3	−10	0.90	<0.001	0.89 to 0.91
4 (Practices with the highest percentage of males)	−13	0.87	<0.001	0.87 to 0.88

**Percentage White**				
(Reference group 1 = practices with the lowest percentage of				
patients from White ethnic group)				
2	24	1.24	<0.001	1.23 to 1.25
3	42	1.42	<0.001	1.41 to 1.44
4 (Practices with the highest percentage of patients from White ethnic group)	36	1.36	<0.001	1.34 to 1.38

**Percentage youngest age group**				
(Reference group 1 = Practices with the lowest percentage of patients aged 15–34 years)				
2	7	1.07	<0.001	1.06 to 1.08
3	12	1.12	<0.001	1.10 to 1.12
4 (Practices with the highest percentage of patients aged 15–34 years)	23	1.23	<0.001	1.21 to 1.25

**Practice size**				
(Reference group 1 = practices with the lowest number of GP-registered patients)				
2	13	1.13	<0.001	1.12 to 1.14
3	26	1.26	<0.001	1.25 to 1.27
4 (Practices with the highest number of GP registered patients)	44	1.44	<0.001	1.42 to 1.45

**Percentage with chronic health illness or disability**				
(Reference group 1 = practices with the lowest percentage of people with chronic health illness or disability)				
2	−1	0.99	<0.001	0.98 to 0.10
3	−2	0.98	<0.001	0.97 to 0.99
4 (Practices with the highest percentage of people with chronic health illness or disability)	−2	0.98	<0.001	0.97 to 0.99

a *Percentage difference calculated using IRR obtained from the negative binomial regression model. For the IMD, the percentage difference represents change across the IMD quintiles in comparison with the reference group IMD Q1 (that is, least deprived practices). For all other variables, the percentage difference represents change across the variable quartiles in comparison with reference group 1 (that is, practices with the lowest population percentage for the given variable). IRR = incident rate ratio. IMD = Index of Multiple Deprivation.*

### NHS App metrics

#### Login sessions

National NHS App login sessions per month ranged from 5157–16 730 430 from January 2019 — May 2021.

**Figure 2.**
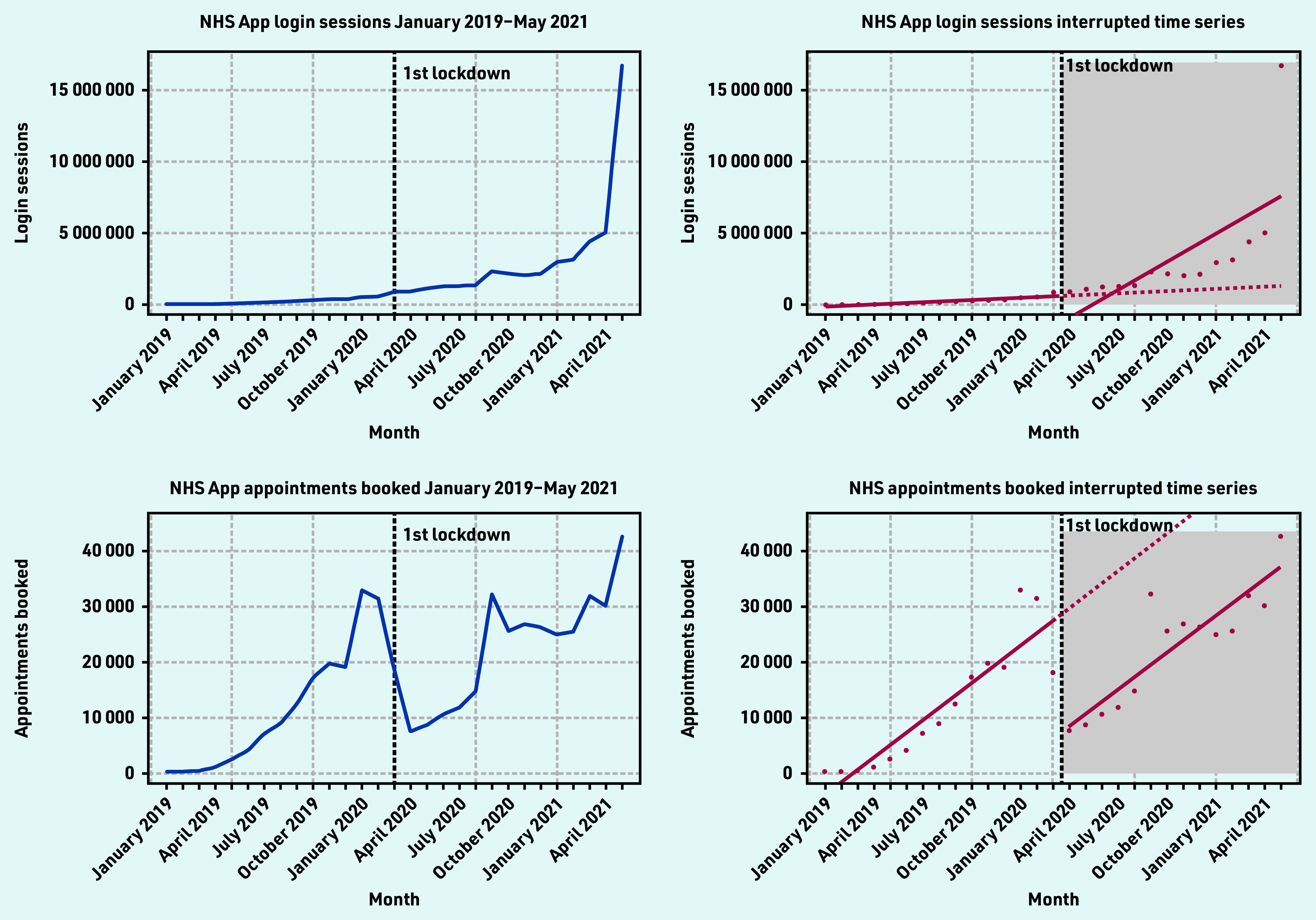
*Login sessions and appointments booked time series and interrupted time series (January 2019 — May 2021).*

There was a steady increase of login sessions from January 2019 — April 2021, with the highest number of logins occurring after introduction of the NHS COVID Pass in May 2021 (16 730 430 login sessions) (Figure 2). The ITS showed that before implementation of the first lockdown, the number of NHS App login sessions was increasing over time at a rate of 52 093 logins a month and decreased immediately after implementation of the first lockdown.

However, only the change in trend over time was found to be positive and significant, indicating an average slope change of 602 124 (*P* = 0.004) logins a month. Twelve months after the first lockdown, the average number of NHS App logins was 5 663 224 more than would have been expected if lockdown did not occur. This represented a 441% increase (Table 2).

**Table 2.** Login sessions and appointments booked interrupted time-series output

**NHS App metric**	**Regression intercept (*P*-value)**	**Pre-intervention monthly trend (*P*-value)**	**Immediate change in trend after COVID-19 lockdown (*P*-value)**	**Change in trend over study period (*P*-value)**	**1 year after first lockdown**	
					**Absolute change (count)**	**Relative change (%)**
Login sessions^a^	−175 074 (0.88)	52 093 (0.68)	−2 164 386 (0.19)	602 124 (0.004)	5 663 224	441
Login sessions^b^	−175 074 (0.36)	52 093 (0.02)	−408 723 (0.1419)	250 991 (*P*<0.001)	2 954 162	222
Appointments booked^a^	−6196 (0.02)	2247(*P*<0.001)	−21 315 (*P*<0.001)	−22 (0.95)	−21 601	−38
Appointments booked^b^	−6196 (0.02)	2247(*P*<0.001)	−20 294 (*P*<0.001)	−226 (0.62)	−23 236	−41

a *January 2019–May 2021.*

b *January 2019–April 2022.*

#### Appointments booked

Appointments booked using the NHS App ranged from 298–42 664 per month during the study period (Table 2 and Figure 2).

There was a surge of appointment bookings in January 2020 (33 003 appointments booked), September 2020 (32 335 appointments booked), and in May 2021 (42 644 appointments booked). There was a significant drop in appointment bookings after the first lockdown was announced in April 2020 (7674 appointments booked).

The ITS showed that before the first lockdown, there was significant evidence of an increase in the average number of appointment bookings 2247 (*P*<0.001), followed by an immediate decrease in bookings after the lockdown was announced −21 315 (*P*<0.001).

Twelve months after the first lockdown, the average number of appointments booked using the NHS App was 21 601 fewer than would have been expected if lockdown did not occur. This represented an overall 38% decrease in appointment bookings.

#### GP health records viewed

**Figure 3.**
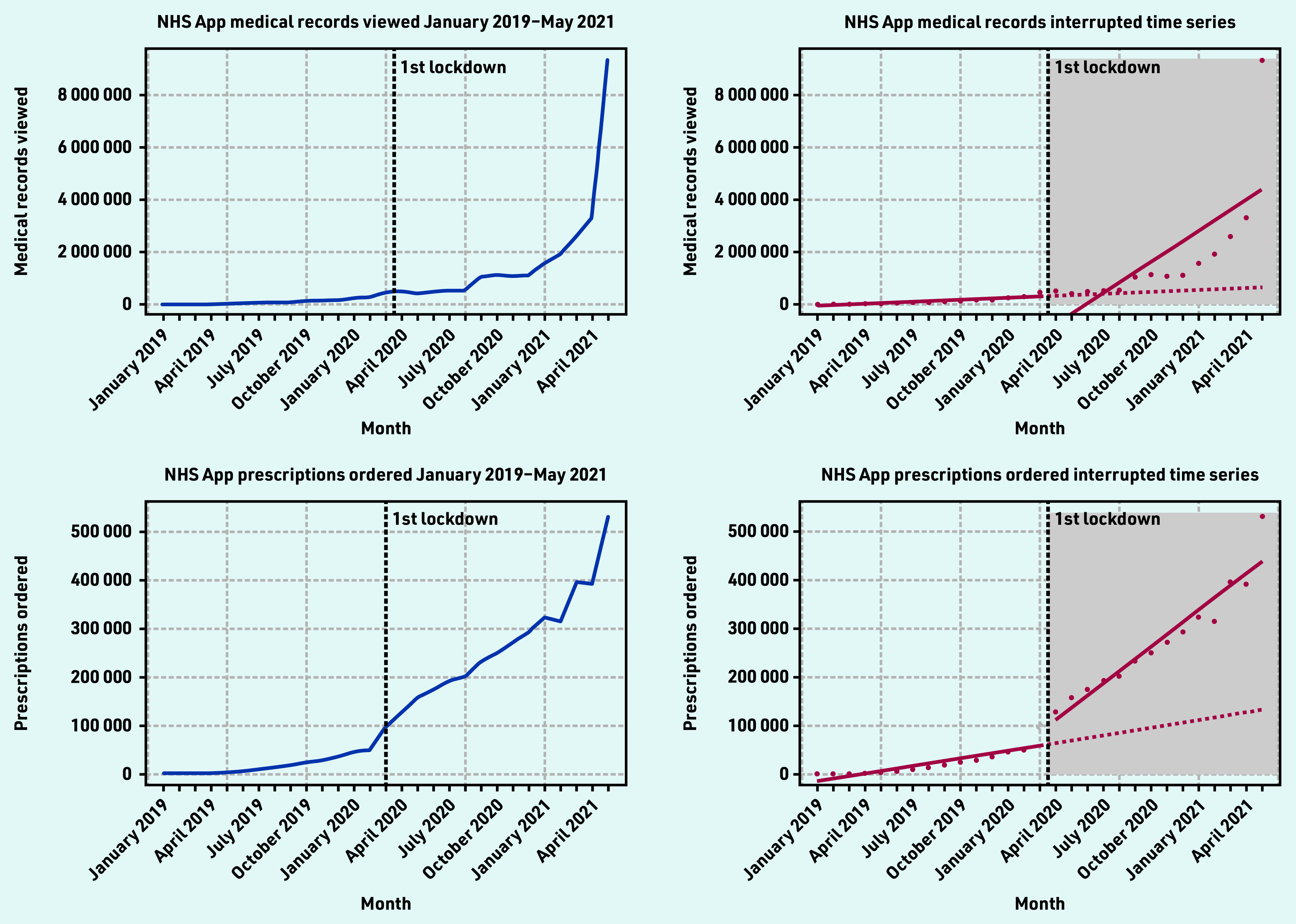
*GP health records viewed and prescriptions ordered time series and interrupted time series (January 2019–May 2021).*

GP health records viewed in the NHS App ranged from 2212–9 324 546 per month from January 2019–May 2021. There was a steady increase in records viewed up until April 2021 with a near three-fold increase from April 2021 (3 309 586 records viewed) and May 2021 (9 324 546 records viewed) (Figure 3). The ITS showed a non-significant decline in the average number of health records viewed right after lockdown −1 441 297 (*P* = 0.11). The sustained effect over time was significant (*P* = 0.001) indicating that the number of records viewed increased on average by 371 656 views per month post-lockdown (Table 3). Twelve months after the first lockdown, the average number of GP health records viewed was 3 390 234 more than would have been expected if lockdown did not occur. This represented a 548% increase.

**Table 3.** GP health records viewed and prescriptions ordered interrupted time-series output

**NHS App metric**	**Regression intercept (*P*-value)**	**Pre-intervention monthly trend (*P*-value)**	**Immediate change in trend after COVID-19 lockdown (*P*-value)**	**Change in trend over study period (*P*-value)**	**1 year after first lockdown**	
					**Absolute change (count)**	**Relative change (%)**
GP health records viewed^a^	−86 104 (0.89)	25 159 (0.72251)	−1 441 297 (0.11)	371 656^d^ (0.001)	3 390 234	548
GP health records viewed^b^	−86 104 (0.56)	25 159 (0.1349)	−495 304^c^ (0.02)	182 458 (*P*<0.001)^e^	1 876 645	303
Prescriptions ordered^a^	−18 935 (0.17)	5225^d^(0.001)	27 494 (0.15)	19 934^e^(*P*<0.001)	286 630	225
Prescriptions ordered^b^	−18 935^c^ (0.01)	5225 ^e^(*P*<0.001)	45 036 (*P*<0.001)	16 425 (*P*<0.001)	258 563	202

a *January 2019–May 2021*.

b *January 2019–April 2021*.

#### Prescriptions ordered

Repeat prescriptions ordered using the NHS App ranged from 655—530 382 per month from January 2019 — May 2021. There was an increase of approximately 1.3 times in prescriptions ordered after the announcement of the first lockdown in March 2020 (98 692 prescriptions ordered) and April 2020 (128 028 prescriptions ordered) (Figure 3). The ITS showed that pre- lockdown, there was significant evidence that the number of prescriptions ordered was increasing at an average rate of 5255 (*P* = 0.001) a month (Table 3).

Post-lockdown, there was a positive and significant increase in trend of 19 934 (*P*<0.001) prescription orders per month (Table 3). Twelve months after the first lockdown, the average number of NHS App prescription orders was 286 630 more than would have been expected if lockdown did not occur. This represented a 225% increase.

## DISCUSSION

### Summary

The study found that there was strong adoption of the NHS App even before the onset of the COVID-19 pandemic. Before the first national lockdown in the UK was announced, there were almost 1.5 million downloads of the app. From April 2021 — May 2021, there was a four-fold increase in app downloads, showing the significant impact the introduction of the NHS COVID Pass had on app uptake. However, a disparity between app downloads and registrations exists. At the end of the study period, there were 8 524 882 downloads and 4 449 869 registrations. There are also differences in the NHS App registration rates across the different sociodemographic groups, highlighting unequal trends of adoption, with higher usage in less deprived and less ethnically diverse practices, with a generally younger population. In terms of app functions, COVID-19 and the introduction of the NHS COVID Pass service had significant impacts on their use. Introduction of the NHS COVID Pass accounted for more than a three-fold increase in login sessions in May, indicating that users could have been logging into the app to retrieve their vaccination status. This pattern was also observed for the number of users using the app to access their GP health records.

Appointment bookings fell substantially after the first lockdown in line with a fall in overall primary care NHS activity,^19^ but continued on the same gradient from March — May 2021. In person GP appointments fell in March 2020 due to the pandemic, and some general practices switched off the ability for patients to book appointments via the NHS App at this time. Prescriptions ordered via the NHS App also significantly increased after the first lockdown, suggesting that more users were utilising the app to place prescription orders rather than in person or via the phone. These findings could be related to patients and carers seeking alternative options to in-person care (for example, digital prescription ordering).^20^ They may have also been influenced by changes in health service delivery owing to the risk of COVID- 19 transmission and because of the reliance on online prescriptions to support patients who had relocated during the national lockdown.^19^

### Strengths and limitations

The ecological analysis was only able to analyse the NHS App at the general-practice level. As person-level data are currently unavailable, these preliminary results indicate an inequality in adoption, which may be influenced by several patient and provider-related factors.

Also, the use of an Interrupted Time Series (ITS) design offers several strengths, allowing for the examination of trends and changes in the use of the NHS App over time, and in relation to COVID-19 timepoints. However, it does not account for other confounding factors, or control for the rapid changes in healthcare delivery during COVID-19, which may have contributed to the results seen in this study. This could include changes in seasonality, other existing co-interventions, and changes in healthcare delivery model that may have occurred in tandem during the study period. The study tried to minimise known confounders by analysing the data with and without the introduction of the NHS COVID Pass, which has been included as Supplementary data.

Furthermore, this study also presents limitations in the use of ecological data to understand subgroup differences in the uptake of and engagement with an app rolled out at a national level. Even though data linking allows some of these challenges to be overcome, differences in the format, structure, and content of different data sources influence biases due to linkage errors. These differences, although potentially negligible, can still result in overestimation or underestimation of results.^21^ Availability of person-level data would overcome some of these challenges by allowing the interactions between sociodemographic factors and app use patterns to be understood more directly.

### Comparison with existing literature

Evidence has suggested that the public health impact of digital health interventions is dependent on real-world uptake, and engagement.^22^ Although the analysis showed a strong interest in the NHS App, variations in the adoption rates and differences in the use of functionalities may not reflect high levels of engagement. A recent study looking at uptake and engagement of health and wellbeing apps found that one of most important factors for engagement were apps coupled with health practitioner support.^22^ At the time of this analysis, the NHS App does not directly incorporate any health practitioner support. This may be an important consideration as research has highlighted the importance of provider endorsement and support, along with technical training and usage assistance as effective digital facilitation strategies in primary care, particularly to support those from marginalised groups.^23^

There are also concerns about sustained app use, as highlighted by the differences in app downloads and registrations, which corroborates the findings of a recent study that showed 25% of all mobile apps were only accessed once after download.^24^ The barriers for sustained app use could be for several reasons, from users not being able to successfully register for the app, or never using the app once it is downloaded, which need to be further explored. Furthermore, the differences in the NHS App registration rates support earlier research findings and they indicate that use of the NHS App, as with other digital health technology, differs according to gender, ethnic group, socioeconomic status, and healthcare needs.^25^^,^^26^

### Implications for research and practice

Further research is needed to identify whether the NHS App is an effective digital health tool and the extent to which it has met the goals set out by NHS England. The existing literature on technology adoption and diffusion of innovation has shown that this process is difficult and complex. Adoption is not just based on the technology, but a complex mixture of how the public and staff interact with it, what they see as the benefits, organisational culture, and wider influences on the system including the policy and regulatory context.^27^ Also, the evidence of usage inequalities warrants facilitation efforts to promote equitable use of digital services in primary care settings. Provider support, patient assistance, and training are identified as useful strategies,^23^ but a more comprehensive understanding of the impact of these initiatives within the broader organisational context, including technology usability and provider burden, is essential.

The analysis showed that there has been strong adoption of the app, and that COVID- 19 has significantly expedited uptake, but further research is needed to evaluate continuous usage of the app over time across different population groups and if it yields any benefits.

In conclusion, this is the first ecological study that has analysed a nationwide intervention rolled out by NHS England. This analysis has shown that the uptake of the NHS App has significantly increased post-lockdown, driven by COVID-19-related events. Usage trends for the different app functions varied and the patterns of app registration was unequal between different subsets of the population. Further research is needed to measure the extent to which it influences inequities in health and whether it impacts care outcomes.

## Data

The authors will make the data used in the publication available on request subject to ethical approvals and institutional data governance arrangements. Additional information relevant to the publication has been provided as supplementary file. The NHS App dashboard can be accessed via https://digital.nhs.uk/services/nhs-app/nhs-app-dashboard.
